# Assessment and indicators of hallux limitus related with quality of life and foot health in school children

**DOI:** 10.3389/fped.2023.1295832

**Published:** 2023-12-13

**Authors:** Claudia Cuevas-Martínez, Ricardo Becerro-de-Bengoa-Vallejo, Marta Elena Losa-Iglesias, Emmanuel Navarro-Flores, Laura Pérez-Palma, João Martiniano, Daniel López-López, Israel Casado-Hernández, Juan Gómez-Salgado

**Affiliations:** ^1^ Research, Health, and Podiatry Group, Department of Health Sciences, Faculty of Nursing and Podiatry, Industrial Campus of Ferrol, Universidade da Coruña, Ferrol, Spain; ^2^ Departament de Podologia, Facultat de Medicina I Ciències de la Salut, Universitat de Barcelona, Barcelona, Spain; ^3^ Facultad de Enfermería, Fisioterapia y Podología, Universidad Complutense de Madrid, Madrid, Spain; ^4^ Faculty of Health Sciences, Universidad Rey Juan Carlos, Alcorcon, Spain; ^5^ Frailty Research Organized Group (FROG), Department of Nursing, Faculty of Nursing and Podiatry, University of Valencia, Valencia, Spain; ^6^ Escola Superior de Saúde da Cruz Vermelha Portuguesa, Lisbon, Portugal; ^7^ Department of Sociology, Social Work and Public Health, Faculty of Labour Sciences, University of Huelva, Huelva, Spain; ^8^ Health and Safety Postgraduate Programme, Universidad Espíritu Santo, Guayaquil, Ecuador

**Keywords:** FHSQ, foot, health related quality of life, functional hallux limitus, hallux limitus

## Abstract

**Background:**

Functional Hallux Limitus (FHL) is a dynamic foot dysfunction characterized by a limitation of hallux dorsiflexion when the first metatarsal head is under load. FHL plays a role in the development of osteoarthrosis in the first metatarsophalangeal joint (IMTPJ). Forefoot disorders can significantly impact an individual's quality of life, leading to dysfunction and pain. The aim of this project was to evaluate the quality of life of school-aged individuals with and without FHL using the Foot Health Status Questionnaire (FHSQ).

**Methods:**

A case-control study was conducted to evaluate the outcomes in paediatric age. A total sample of 116 children between 6 and 12 years old was used to conduct this research. The sample was divided into two groups: (i) the healthy group (*n *= 58) and the FHL group (*n* = 58). The FHSQ was completed and the FHL test was performed in a seated position to classify the patients into the selected group.

**Results:**

Non-significant changes were observed when the mean values of the FHSQ domains were compared between the groups with and without FHL, except for the “general foot health” domain (*p *= 0,024) associated with the specific foot health section (section 1) of the Questionnaire. For the domains linked with the general well-being section (section 2), there was not a statistically difference in the mean of the scores obtained between the two school-aged groups with and without FHL, being slightly lower in the group with the presence of FHL for the overall health and physical function domains. Both the healthy and case groups obtained and identical range of scores (10–100) for the “foot pain” domain. Nevertheless, the mean of the score was lower for the participants with FHL.

**Conclusions:**

The perception of the quality of general foot health was poorer in the school-aged group with FHL. Variables such as foot pain and footwear are likely contributors influencing the perception of foot health quality. The school-aged population with FHL faces a decline in the quality of foot life. Ensuring adequate foot control in children and implementing future foot programs for this population are imperative for enhancing school children's perception of foot health and managing the development of pain and footwear-related issues.

## 1. Introduction

Functional hallux limitus (FHL) pathology is described as a functional inability of the proximal phalanx of the hallux to perform the dorsiflexion movement (DF) during the complete gait cycle and the final phase of propulsion (1, 2). Therefore, this impairment only occurs in a closed kinetic chain. In an advanced limitation phase of the first metatarsophalangeal joint (IMTPJ) and mainly in adulthood, FHL can develop and degenerate into hallux limitus, followed by hallux rigidus and even hallux valgus (3). Joint osteoarthrosis is very common in adulthood, being present in 10% of people between the ages of 20 and 34 years and increasing in incidence proportionally with age (3). When this limitation appears in both closed and open kinetic chains, it is called hallux limitus and is the previous step to culminating in total restriction of movement in this joint, called hallux rigidus (4).

Forefoot disorders can deteriorate the quality of life of individuals causing dysfunction and even pain (4) but the incidence and prevalence of FHL remain unclear and very poorly understood (1).

In the initial approach, the foot has important functions for cushioning and transmitting ground reaction forces when standing and throughout the whole gait cycle. FHL is fundamental to the study of foot biomechanics in the sagittal plane. Dananberg described in his sagittal plane theory that a block in any joint that generates a movement in this plane will cause a compensation in another joint that may be located above or below the affected joint (5). A biomechanical decompensation that generates high plantar fascia tension, for example, will result in greater opposing forces between the downward vertical forces of the plantar flexion motion of the first metatarsal head compared to the upward vertical forces of hallux extension, causing a limitation or restriction of the IMTPJ DF motion (2). A decreased DF range of the IMTPJ causes insufficient physiological locking of the lateral column of the foot and hinders the correct advancement of forces and center of mass during dynamics, in addition to an insufficient windlass mechanism. Consequently, the foot will be unstable during the stance phase and the IMTPJ pivot during the propulsive phase will be ineffective (2).

Based on these antecedents, it is necessary to study the effects of the presence or absence of FHL at school age, to know if at early ages with a functional limitation of the IMTPJ, quality of life can be negatively affected and to avoid, as far as possible, greater future affectations, such as osteoarthrosis, among other biomechanical compensations that cause pain and secondary pathologies.

Finally, our hypothesis was that school children with FHL would show poor values associated with foot health and quality of life in comparison with school children without FHL. Thus, the aim of this project was to evaluate the quality of life in school-aged individuals with and without FHL using the FHSQ.

## 2. Material and methods

### 2.1. Participants

A case control study was carried out in multiple schools from different places in Catalonia (Spain) and was developed between January 2022 and February 2023. The sampling method was consecutive to recruit 116 children aged between 6 and 12 years with a mean ± SD (9.55 ± 1.54) years. This study was performed consecutively and conveniently.

This case control study followed all the criteria of the Strengthening the Reporting of Observational Studies in Epidemiology (STROBE) guidelines (6) assessed FHSQ results in school-aged children with and without functional hallux limitus.

For this project, all subjects met the inclusion criteria to be part of the study and did not show any of the exclusion criteria.

The following inclusion criteria for both groups of participants were met: (1) age between 6 and 12 years; (2) absence of musculoskeletal disorders or significant general conditions; (3) absence of previous surgical treatment or trauma to the lower extremities; (4) having flexible feet; (5) participants with and without FHL; (6) parents and children who agreed to complete the FHSQ, (7) legal guardians who agreed to sign the written informed consent, and (8) subjects interested in participating and completing all phases of the study.

The exclusion criteria were: (1) age not between 6 and 12 years; (2) patients under treatment with any medication that could affect the final results; (3) presence of musculoskeletal disorders or neurological disease; (4) hypermobility syndrome; (5) participants with a IMTPJ angular value of less than 10° with knee extension and with an ankle angular value of less than 10° with the knee extended; and 6) subjects who refused to comply with the guidelines for participating in the study.

The research was approved by the Human Ethics Committee of the Universitat de Barcelona, Barcelona, Spain (Ethics Code: IRB 00003099). All actions complied with all current regulations on human experimentation, as well as the Declaration of Helsinki and Organic Law 3/2018, of December 5, on protection of personal data and guarantee of digital rights (7).

### 2.2. Procedure

The study was performed by an expert podiatrist with more than 10 years' experience in biomechanical evaluation.

The legal documentation and FHSQ were given to the volunteers' legal guardians interested in participating in the study, to be read and completed calmly at home.

The FHSQ is a foot health status questionnaire and has been recognized as a validated tool (8). Section 1 is associated with specific foot health and consists of 13 questions reflecting four foot health-related domains: pain, function, footwear, and general foot health. For each section, different questions are answered with specific words (“none”, “very mild”, “mild”, “moderate”, “severe”). The first section has demonstrated a high degree of content, criterion, and construct validity (Cronbach *α *= 0.89–0.95) and high retest reliability (intraclass correlation coefficient = 0.74–0.92). The section 2 is linked with general well-being and includes questions that reflect four general health-related domains: general health, physical activity, social capacity and vigour. The domains and questions in this section are largely adapted from the Medical Outcomes Study 36-Item Short-Form Health Survey, which has been validated for use in the Spanish population (9).

The day of sample collection, participants came to the designated location, carrying the necessary documentation described in the inclusion criteria. Subsequently, they removed their shoes and socks. They were then measured and weighed by the clinician to ensure that the anthropometric measurements were current. Once these data were obtained, the body mass index (BMI) was calculated, and the participating subjects were checked to ensure that they did not meet any exclusion criteria.

Next, the clinician checked the range of motion of the IMTPJ and ankle DF to discard hallux rigidus (HR) and ankle equinus. To assess this, the patient was in a seated position and the subtalar joint was in a neutral position, then maximum DF of the ankle was performed with the knee extended. To verify that the DF of the IMTPJ was greater than 10°, it was measured with an arm goniometer (10).

After selecting the participants who met all the study inclusion criteria, they completed the FHL test described by Dananberg and scientifically validated (11), and were classified into the corresponding group according to the presence or absence of FHL. To perform the FHL test, the subject is placed in a seated position, barefoot and without socks so that the clinician can perform the test. The test consists of holding the foot with one hand and placing the thumb under the first metatarsal head. Then, with the contralateral hand, pressure is exerted on the proximal phalanx of the first toe to perform DF of the IMTPJ. If both forces are similar, the result for the FHL test is negative (FHL−), whereas if the force for IMTPJ DF is greater than that exerted under the first metatarsal head, it is considered a positive result (FHL+).

Data acquisition from the FHSQ was performed by entering the score for each individual and for each specific question into the software (The Foot Health Status Questionnaire, Version 1.03), which transforms the raw scores and sums them into sections. Scores range from 0 to 100, with 0 being the worst value and 100 the best value. In addition, the software provides graphical illustrations of the results (8, 12). To facilitate the response and adapt it to the subjects participating in this study, the FHSQ was translated into Spanish (13).

The outcome measurements for subjects diagnosed with functional hallux limitus and healthy matched-paired controls included foot pain, foot function, footwear, general foot health, overall health, physical function, social capacity, and vigor.

To calculate the sample size for this study, the specific levels of confidence, power and equal size groups were applied using the Epidat software version 4.2 created by public organizations and aimed at epidemiologists and other health professionals that analyze tabulated data (14). To achieve statistical confidence, a statistical power of 80% with a *β* error of 20%, an *α* error of 0.05 and a two-tailed test were established. A total sample of 116 school-aged children aged between six and twelve years were included in the study and divided into two groups of 58 subjects each. One group had FHL and the other did not.

Statistical analyses were carried out with the Statistical Package for the Social Sciences (SPSS software, version 19.0). Parametric data were described as normal mean, standard deviation (SD) and range (minimum-maximum values). Normality was tested with the Kolmogorov–Smirnov test for the variables studied (*p* > 0.05) in the data on FHSQ results. Independent *t*-tests were used for outcome variables that were normally distributed. The non-parametric Mann–Whitney “*U*” test was performed to consider contrasts between the two groups with or without FHL.

In all analyses, a statistically significant result was considered when *p* < 0.05 (with 95% confidence interval).

## 3. Results

Out of the 116 participants recruited for this study, a total of 58 were diagnosed with FHL and the other 58 were healthy. The total sample consisted of 54 boys and 62 girls, and no significant results were observed between the two groups based on quantitative sociodemographic and descriptive data (Table 1).

**Table 1:** Quantitative sociodemographic and descriptive data for patients diagnosed with functional hallux limitus, healthy controls and total sample.

Quantitative descriptive data	Total group (*n* = 116) Mean ± SD (Range)	FHL (*n* = 58) Mean ± SD (Range)	Healthy (*n* = 58) Mean ± SD (Range)	*p*-Value
Age (years)	9.55 ± 1.54 (6.00–12.00)	9.72 ± 1.36 (7.00–12.00)	9.38 ± 1.69 (6.00–12.00)	0.302^a^
Weight (Kg)	36.73 ± 11.40 (17.95–90.00)	36.72 ± 9.53 (20.00–66.00)	36.74 ± 13.09 (17.95–90.00)	0.515^a^
Height (cm)	140.64 ± 11.03 (113.00–176.00)	140.90 ± 9.78 (120.00–167.00)	140.40 ± 12.22 (113.00–176.00)	0.730^a^
BMI (kg/m^2^)	18.29 ± 4.01 (11.00–40.00)	18.21 ± 3.26 (14.00–30.00)	18.38 ± 4.67 (11.00–40.00)	0.764^a^
Sex (male/female)	54/62 (46.60/53.40)	24/34 (41.40/58.60)	30/28 (51.70/48.30)	0.264^b^
Foot size	35.86 ± 3.35 (15.00–43.0)	36.22 ± 2.34 (30.00–43.00)	35.50 ± 4.11 (15.00–42.00)	0.418^a^

Kg, kilogram; Cm, centimeter; m^2^, square meter; % Percentage; SD, standard deviation; N, number.

^a^ Mann–Whitney *U*-test was used.

^b^ Fisher exact test was used. In all the analyses, *p* < 0.05 (with a 95% confidence interval) was considered statistically significant.

The values obtained were not significant to determine differences between the study groups. This indicates that there was no relationship between the presence or absence of FHL and weight, height, BMI, sex, or foot size among the study sample aged 6–12 years used for this project.

Table 2 shows the relationship between the population samples studied and the FHSQ results obtained for each domain. Section 1 is associated with specific foot health outcomes: foot pain, foot function, general foot health, and footwear showing significant differences (*p* < 0.05) for general foot health. They showed a worse QoL related to foot health for FHL, with the FHL group having lower scores than children without FHL, but not significant differences for foot pain, foot function and footwear (*p* > 0.05). The section 2 is linked with general well-being assesses four domains without showing significant differences (*p* < 0.05) for overall health, physical function, social capacity, and vigor.

**Table 2:** The relation between positive and negative FHL patients and FHSQ scores.

	Total group (*n* = 116) Mean ± SD (Range)	FHL (*n* = 58) Mean ± SD (Range)	Healthy (*n* = 58) Mean ± SD (Range)	*p*-Value
1. Specific foot health				
Foot Pain	90.26 ± 16.15 (10.00–100)	88.15 ± 17.99 (10.00–100)	92.37 ± 13.90 (10.00–100)	0.285^a^
Foot function	94.02 ± 12.62 (43.75–100)	92.56 ± 14.22 (43.75–100)	95.47 ± 10.72 (43.75–100)	0.098^a^
Footwear	60.42 ± 17.68 (8.33–75)	58.48 ± 18.37 (16.67–75.00)	62.36 ± 16.90 (8.33–75)	0.285^a^
General foot health	78.45 ± 21.07 (25–100)	73.14 ± 23.87 (25–100)	83.75 ± 16.43 (25–100)	0.024^a^
2. General well-being				
Overall health	84.91 ± 18.90 (30–100)	84.31 ± 17.58 (30–100)	85.52 ± 20.28 (30–100)	0.255^a^
Physical function	94.88 ± 11.88 (22.22–100)	94.44 ± 10.96 (55.56–100)	95.31 ± 12.82 (22.22–100)	0.254^a^
Social capacity	93.32 ± 14.03 (25–100)	94.61 ± 13.87 (25–100)	92.03 ± 14.18 (37.50–100)	0.186^a^
Vigor	78.40 ± 17.30 (25–100)	79.53 ± 16.79 (25–100)	77.26 ± 17.86 (37.50–100)	0.515^a^

SD, standard deviation; N, number.

In all the analyses, *p* < 0.05 (with a 95% confidence interval) was considered statistically significant.

^a^ Mann–Whitney *U*-test was used.

## 4. Discussion

This study contributes to a greater understanding of the influence of FHL on the quality of life of the school-age population by comparing self-reported FHSQ scores of a sample of 116 school-aged individuals. A decrease in the general score of the FHSQ would mean that FHL is certainly affecting foot health at this early stage. Utilising research for health professionals to advance in their practice will ensure appropriate knowledge and will provide high-quality advice and care to this target population in the future. However, to the best of the authors' knowledge, the lack of epidemiological studies addressing FHL makes it challenging to accurately assess the impact of this condition on school children.

A comparison of the mean scores obtained in the FHSQ between both groups revealed a relationship between the perception of general foot health and the presence of FHL. This lower perception of general foot health seems to be related to the lower scores obtained for foot pain and the footwear domain. Similar observations were addressed in the study performed by López_López et al. (15) in school-aged children to determine whether arch height has an effect on the health-related quality of life. The study reported that the children experience foot pain, restrictions in terms of footwear and, in general, a worse state of foot health. Nevertheless, they couldn't establish a relationship between these findings and the height arch which differs from this study where the score of the general foot health domain was directly related to the presence of FHL.

The outcomes of this investigation are aligned with findings shown in populations experiencing foot problems associated with the first MTPJ, particularly in more advanced stages, characterized by progressive subluxation and osteoarthritis. Lazarides et al. (16) and López et al. (17) have suggested that disorders involving the first MTPJ, such as hallux valgus or hallux rigidus may have an adverse impact on the perception of general foot health during adulthood and among older individuals when compared to the earlier stages such as in FHL dysfunction. Consequently, effective management of alterations in the first MPTJ during the initial stages can potentially embrace a positive influence on the status and perception of general foot health in the later life stages which is aligned with the current study perspectives. Similarly, the presence of hallux valgus has been correlated with a lower quality of life, increased foot pain, disability, and functional limitations, as demonstrated in a study performed by Gonzalez-Martin et al. (18) involving a random population sample of 1,837 individuals in Spain. Furthermore, Gilheany et al. (3) examined foot health among adult patients with hallux valgus and hallux rigidus, who were candidates for surgery to address these conditions. The Foot Health Status Questionnaire (FHSQ) was administered both pre- and post-surgery. The hallux valgus group exhibited consistently low scores in areas of pain, foot function, shoe fit, and overall foot health. Notably, individuals with hallux rigidus scored even lower, indicating that as the motion of the first metatarsophalangeal joint becomes severely limited, general foot health markedly deteriorates. In contrast to the outcomes of this study, it is evident that hallux valgus and hallux rigidus conditions affect in depth of the first metatarsophalangeal joint (MTPJ), leading to tissue degeneration and a decline in overall quality of life.

To mitigate potential significant biases in this study, the participants presenting generalized ligamentous laxity were excluded so this condition was adopted as an exclusion criterion. This condition is common within the school-age population and is characterized by excessive joint mobility associated, among others, with foot pain and several foot disorders. The resolution of exclusion was based on the results of the study conducted by Palomo-López et al. (19) after analysing a sample of 100 participants from 18 to 35 years old with and without general ligamentous laxity and the correlation of this condition with FHSQ domains. The results suggested that general ligamentous laxity is related to more foot pain, greater restrictions in terms of footwear and a worse state of foot health thus, it was considered as an uncontrolled condition that could affect the interpretation of the results of the current study. The present study had some limitations such as including a larger and a diverse sample of individuals from various countries or even different country regions. Including different ethnicities would make a worthwhile contribution to the strength of this research and may help to identify if there is a cultural difference related to FHL disease in terms of pain, footwear, foot health perception and well-being thus, future research would be beneficial for improving the knowledge in this field and target population once addressing these limitations.

## 5. Conclusions

The perception of the quality of general foot health was poorer in the school-aged group with FHL. Variables such as foot pain and footwear are likely contributors influencing the perception of foot health quality. The school-aged population with FHL faces a decline in the quality of foot life. Ensuring adequate foot control in children and implementing future foot programs for this population are imperative for enhancing school children's perception of foot health and managing the development of pain and footwear-related issues.

## Data Availability

The raw data supporting the conclusions of this article will be made available by the authors, without undue reservation.
